# Zinc-Ion Storage Mechanism of Polyaniline for Rechargeable Aqueous Zinc-Ion Batteries

**DOI:** 10.3390/nano12091438

**Published:** 2022-04-23

**Authors:** Jiangfeng Gong, Hao Li, Kaixiao Zhang, Zhupeng Zhang, Jie Cao, Zhibin Shao, Chunmei Tang, Shaojie Fu, Qianjin Wang, Xiang Wu

**Affiliations:** 1 College of Science, Hohai University, Nanjing 210098, China; lihao5799@163.com (H.L.); kxzhang@126.com (K.Z.); zzp18752006001@163.com (Z.Z.); caojie@hhu.edu.cn (J.C.); zbshao@hhu.edu.cn (Z.S.); 2 National Laboratory of Microstructures, Nanjing University, Nanjing 210093, China; fushaojie@nju.edu.cn (S.F.); qjwang@nju.edu.cn (Q.W.); 3 School of Materials Science and Engineering, Shenyang University of Technology, Shenyang 110870, China

**Keywords:** zinc-ion batteries, conducting polymers, polyaniline, zinc-ion diffusion

## Abstract

Aqueous multivalent ion batteries, especially aqueous zinc-ion batteries (ZIBs), have promising energy storage application due to their unique merits of safety, high ionic conductivity, and high gravimetric energy density. To improve their electrochemical performance, polyaniline (PANI) is often chosen to suppress cathode dissolution. Herein, this work focuses on the zinc ion storage behavior of a PANI cathode. The energy storage mechanism of PANI is associated with four types of protonated/non-protonated amine or imine. The PANI cathode achieves a high capacity of 74 mAh g^−1^ at 0.3 A g^−1^ and maintains 48.4% of its initial discharge capacity after 1000 cycles. It also demonstrates an ultrahigh diffusion coefficient of 6.25 × 10^−9^~7.82 × 10^−8^ cm^−2^ s^−1^ during discharging and 7.69 × 10^−10^~1.81 × 10^−7^ cm^−2^ s^−1^ during charging processes, which is one or two orders of magnitude higher than other reported studies. This work sheds a light on developing PANI-composited cathodes in rechargeable aqueous ZIBs energy storage devices.

## 1. Introduction

To build a low-carbon society, green energy sources such as solar energy and wind energy were developed rapidly. A challenge exists in terms of how we can adapt these intermittency renewables to the electricity grid. Thus, it is essential to develop large-scale electrochemical energy storage technologies. In recent years, much effort has been focused on aqueous multivalent ion batteries (zinc-ion batteries (ZIBs) [1,2], magnesium-ion batteries [3], calcium-ion batteries [4], and aluminum-ion batteries [5]) according to the following reasons: (1) The aqueous electrolytes are much safer than flammable organic electrolytes. (2) The ionic conductivity of aqueous electrolytes (~1 S cm^−1^) is much higher than that of organic electrolytes (~1−10 mS cm^−1^), which enable a fast intercalation/de-intercalation rate. (3) During charge/discharge processes, multivalent ions enable more than one electron transfer, which imply that multivalent ion batteries can offer high gravimetric energy densities.

Exploring high-performance electrode active materials is a critical factor to construct advanced energy storage batteries. Until now, a variety of active materials has been developed and assembled as rechargeable batteries [6,7,8,9]. During long cyclic usage, the electrochemical performance of the assembled rechargeable batteries inevitably shows degradation, and the reliability of the batteries is seriously limited. J.W. Wang et al. studied the lithiation/delithiation of micro-sized Sn particles using the in situ transmission electron microscopy technique; the results demonstrated that degradation is attributed to particle pulverization generated by the lithiation-induced, large and inhomogeneous volume changes [10]. Similar conclusions were demonstrated by Y. Sun in pulverized V_2_O_5_ powder [11]. To overcome the capacity fade originating from irreversible phase conversion and structure dissolution, a conductive polymer was often chosen by researchers to suppress cathode dissolution. Among the family of conductive polymers, polyaniline (PANI) was the most popular media because of its high conductivity and reversible electrochemical response during anodic oxidation and cathodic reduction. For example, J. H. Huang et al. designed a polyaniline-intercalated-layered MnO_2_, in which the PANI polymer eliminated phase change and alleviated volume change upon cation insertion/extraction [12]. W.J. Li et al. designed a vanadium oxide (V_2_O_5−x_)/PANI superlattice to strengthen the alternative layered structure, where the PANI layer restrains the dissolution of V_2_O_5−x_ active materials in aqueous electrolytes, which worked as structural stabilizer, enabling a high-rate capability and a long-term cycling life [13]. The PANI-GO/CNT cathode and PANI-intercalated VOH were also widely reported [14,15]. In such energy storage systems, PANI jumbles with host materials, which show the synergistic energy storage effect. Despite reports showing high specific capacity, the reasons for the improved performance after adding PANI remain ambiguous and need to be further explored; in particular, there is a lack of comprehensive studies on the charge storage mechanism and ion transport kinetics of PANI.

In view of the attractive properties of aqueous multivalent ion batteries, we investigated the electrochemical performance and ion transport kinetics of PANI cathode to further understand Zn^2+^ storage mechanisms. It was found that the charge/discharge processes of PANI can be controlled by protonation, and it is associated with four types of nitrogen, including non-protonated amine −NH−, protonated amine −NH^+^−, non-protonated imine −N=, and protonated imine −NH^+^=. The assembled PANI/Zn cell achieves a high capacity of 74 mAh g^−1^ at 0.3 A g^−1^ and maintains 48.4% of its initial discharge capacity after 1000 cycles. Importantly, the Zn^2+^ diffusion coefficient in the PANI cathode is within the range of 6.25 × 10^−9^ to 7.82 × 10^−8^ cm^−2^ s^−1^ for discharge processes and 7.69 × 10^−10^ to 1.81 × 10^−7^ cm^−2^ s^−1^ for charge processes, which is one or two orders of magnitude higher than any other reported cathode materials for ZIBs [11,12,13,16,17,18]. Our findings herein will inspire the modification of PANI-intercalated cathode materials for high performance ZIBs.

## 2. Materials and Methods

### 2.1. Chemical Reagents

All chemical reagents were of analytical grade and were used as received without further purification. Sulphuric acid (H_2_SO_4_, 98%) and aniline (99.5%) were purchased from Chengdu Kelong Chemical Reagent Co. (Chengdu, China). Stainless steel films and zinc foil were purchased from Guangdong Canrd New Energy Technology Co., Ltd. (Dongguan, China). All solutions were prepared with deionized water.

### 2.2. Materials Preparation

PANI films were anodically electrodeposited by cyclic voltametric (CV) methods on an electrochemical workstation (CHI660E, Chenhua, Shanghai, China). Saturated calomel electrode (SCE, the potential vs. SHE is 199 mV) and platinum sheets were used as the reference electrode and counter electrode, respectively. After cleaning by plasma bombardment to optimize hydrophilicity, the stainless-steel substrates were carefully coated with a thick film Polyvinyl chloride (PVC) with an exposed surface area of 1.54 cm^2^. The electrolyte was prepared by dropping 2.72 mL H_2_SO_4_ into 200 mL 2 M aniline solution with vigorous stirring until obtaining a clear brown solution. The PANI film was electroplated at a scan rate of 25 mV s^−1^ for 30 cycles ranging from −0.1 to 0.9 V. After deposition, the as-prepared PANI film was carefully washed with distilled water to remove unreacted aniline and dried out in a drying cabinet. The mass loading of active material was around 1.0 mg cm^−2^

### 2.3. Physicochemical Characterizations

An X-ray diffractometer (XRD, D8 ADVANCE, Bruker, Karlsruhe, Germany) using Cu Kα radiation (λ = 1.5418 Å) was used to analyze the phases and structures of the deposited films. A scanning electron microscope (SEM, Quanta 200, FEI, Hillsborough, OR, USA) was used to study the morphologies and microstructures of the samples. Transmission electron microscopy (TEM, Tecnai F20, FEI, Hillsborough, OR, USA) and high-resolution TEM images were taken to confirm the size as well as the crystalline structure of the PANI film. Integrated elemental compositions over an area was collected using energy dispersive X-ray spectroscopy (EDS GENESIS Apex, EDAX Inc. Mahwah, NJ, USA) equipped with TEM. X-ray photoelectron spectroscopy (XPS ESCALAB 250 Xi, Thermo Fisher Scientific, Waltham, MA, USA) measurements were performed by using a monochromatic Al Kα X-ray beam (1486.6 eV), The binding energies were calibrated using C 1s peak (BE = 284.6 eV) as a standard.

### 2.4. Electrochemical Measurements

The PANI/Zn batteries were assembled using PANI film with stainless-steel substrates as the cathode, Zn foil (diameter: 15.6 mm, thickness: 50 μm) as the anode, and Whatman glass fiber as the separator in CR2032 coin cells. A 2 M quantity of Zn (CF_3_SO_3_)_2_ was used as the aqueous electrolyte. All cells were assembled in the ambient environment. The electrochemical performance measurements were performed by a multichannel battery testing system (CT-4008, Neware, Shenzhen, China) with a voltage window of 0.3–1.8 V (vs. Zn^2+^/Zn) at 20 °C. The specific capacity was calculated based on the mass of PANI in cathode. CV curves were collected on an electrochemical workstation (CHI660, Chenhua, Shanghai, China) within the same voltage window at different scan rates from 0.1 to 1 mV s^−1^. The electrochemical impedance spectra (EIS) were performed in a frequency range of 10^−2^~10^5^ Hz with an AC voltage amplitude of 5 mV (CHI660, Chenhua, Shanghai, China).

## 3. Results

**Figure 1:**
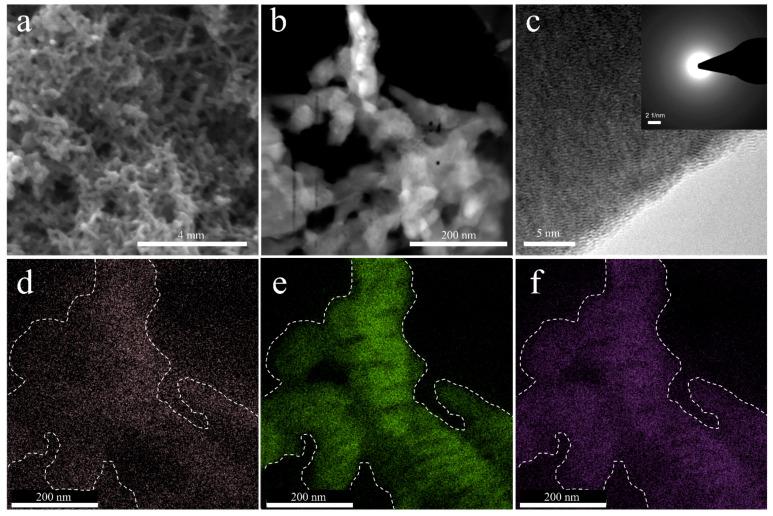
(**a**) SEM, (**b**) TEM, and (**c**) high-resolution TEM images of PANI film. The inset of (**c**) shows the corresponding SAED image. (**d**–**f**) Corresponding EDS mapping of the nitrogen (N), oxygen (O), and sulfur (S) in PANI film.

The PANI electrode was prepared on stainless steel through a facile electrodeposition method. During the electrochemical polymerization process, aniline monomers polymerized and formed long-chain PANI. The typical microstructures of PANI are presented in Figure 1a, which shows a continuous three-dimensional network. The pure PANI film shows short rods clusters with diameters of ~50 nm and lengths of 150–200 nm (Figure 1b), which can provide enough electrochemical active sites for adsorbing ions. The high-resolution TEM image in Figure 1c shows their short worm-like characterization, and the selected area electron diffraction (SAED) of PANI (inset of Figure 1c) presents dispersed diffraction rings, which illustrates the amorphous character of the sample. The XRD pattern (Supplementary Figure S1) shows the amorphous nature of PANI. However, some signals are launched at 2*θ* = 6.3°. The signal is assigned as the periodicity distance between the dopant and N atom on adjacent main chains [19,20]. Figure 1d–f shows the EDS mapping of the PANI film, and the dashed line shows the outline of Figure 1b. The elements of N, O, and S are distributed uniformly, implying the homogenous doping of SO_4_^2−^ in the polyaniline’s long chain.

**Figure 2:**
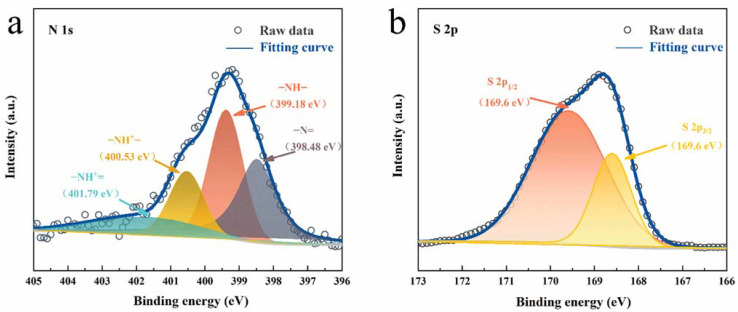
Core level XPS of (**a**) N 1s and (**b**) S 2p.

XPS was also carried out to characterize the valence states and chemical composition of PANI film. The survey XPS scans of the PANI films indicate the presence of sulfur (S 2p, 168.85 eV), carbon (C 1s, 286.32 eV), nitrogen (N 1s, 401.07 eV), and oxygen (O 1s, 533.21 eV), as shown in Supplementary Figure S2. C and N are expected to originate from PANI film, while S may derive from H_2_SO_4_ in electrochemical solutions. The N 1s core level XPS spectrum can be deconvoluted into four peaks, as shown in Figure 2a. The peak at 398.48 eV corresponds to −N= (quinoid imine), the main peak at 399.38 eV is ascribed to −NH− (benzenoid amine), and the two remaining peaks located at 400.53 and 401.79 eV may be attributed to protonated nitrogen −NH^+^− and −NH^+^= [21]. Moreover, the XPS analysis of S 2p can be deconvoluted into S 2p_1/2_ (169.6eV) and S 2p_3/2_ (168.6eV) in Figure 2b, and the S 2p peak is fitted with the spin-orbit doublets of sulfate groups. The doped SO_4_^2−^ remaining in PANI’s long chain could play the role of rapid balance charges during redox reactions [22]. The morphological and structural advantages of the PANI cathode described above are favorable for ion diffusion and Zn ion storage during charge/discharge.

**Figure 3:**
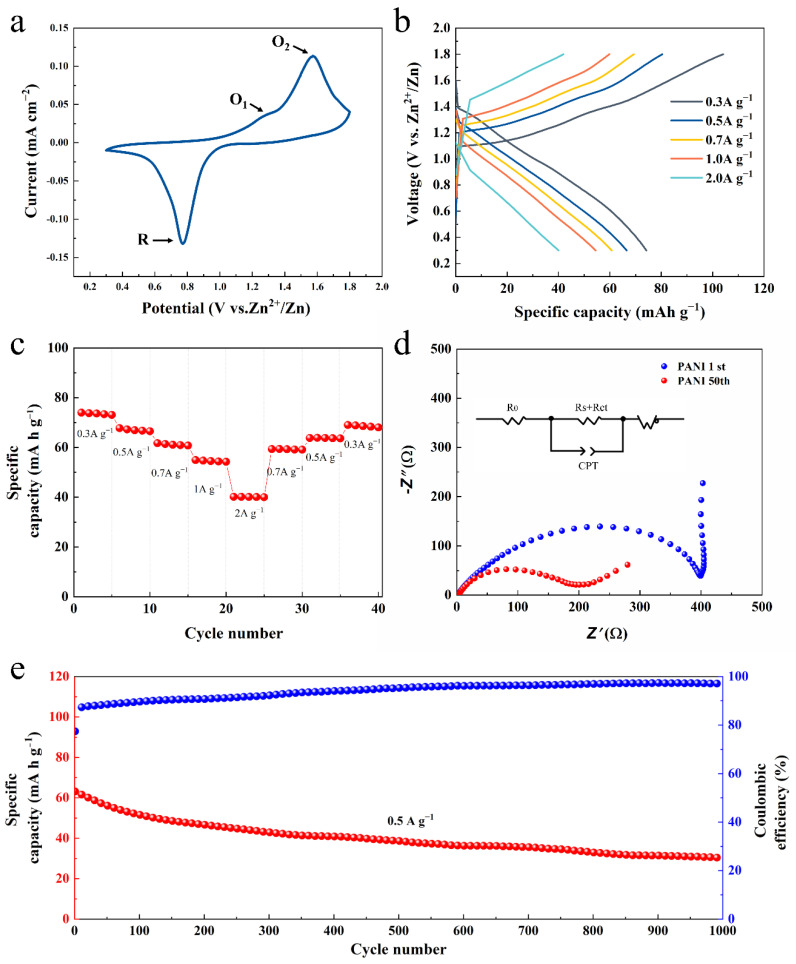
Electrochemical performance of PANI electrode. (**a**) CV curve measured at 0.1 mV s^−1^. (**b**) Typical galvanostatic charge/discharge curves scanned from 0.3 A g^−1^ to 2 A g^−1^. (**c**) Rate performance with the charge/discharge current densities varying from 300 to 2000 mA g^−1^. (**d**) EIS Nyquist plots and (**e**) cycling performance of the cell.

The electrochemical profile is characterized in the typical 2032 cell. Figure 3a shows CV curve tested at 0.1 mV s^−1^ with the potential window of 0.3~1.8 V. There is one pair of cathodic peaks (R and O_2_ marked in Figure 3a) and one small shoulder (O_1_) next to the O_2_ peak. These represent the reduction/oxidation process during adsorption/desorption of Zn^2+^. The galvanostatic discharge–charge curves of PANI in Figure 3b show a steeper slope, especially at large current densities and a high discharge capacity of 74 mA h g^−1^ at 0.3 A g^−^^1^. The rapid charge–discharge speed corresponds to the fast ion absorb–desorption and redox reaction. In rate capability tests (Figure 3c), the PANI electrode with a mass loading of 1 mg cm^−2^ delivers a relatively stable capacity. With an increase in current density from 0.3 to 0.5, 0.7, 1, and 2 A g^−1^, the cell delivers specific capacities of 68, 68, 58, and 40 mA h g^−1^, respectively. When the current densities decrease back to 0.3 A g^−1^ from 2 A g^−1^, the capacities recover to the initial values, suggesting a stable structure and great electrochemical reversibility. The EIS spectra and the equivalent circuit model of PANI are presented in Figure 3d. The impedance measurements are taken after discharging at the 1st cycle and 50th cycle. Both spectra comprised a semicircle in the high frequency region, which originated from the solid/electrolyte interfacial resistance. The interception between semicircle and the real axis corresponds to the migrating resistance of Zn^2+^ ions through the surface layer (R_s_), and the semicircle represents the charge transfer resistance (Rct) [23]. The Rct value transformed from 398.2 Ω to 195.5 Ω after cycling, which might be attributed to the activation of materials. While at the low frequency region, the inclined line is caused by the Zn^2+^ ions’ chemical diffusion impedance (Warburg impedance). The result of EIS further demonstrates that the PANI film with amorphous nature effectively enhances the electrochemical kinetics by decreasing the impedances. As shown in Figure 3e, the 3D conductive network PANI ZIBs could maintain a discharge capacity of 30 mAh g^−1^ (48.4% of its initial discharge capacity) after 1000 cycles with high Coulombic efficiency close to 100%. The polymer retains the 3D network’s morphology without being peeled off from the substrate. Such high stability of the electrode guarantees excellent capacity retention.

**Figure 4:**
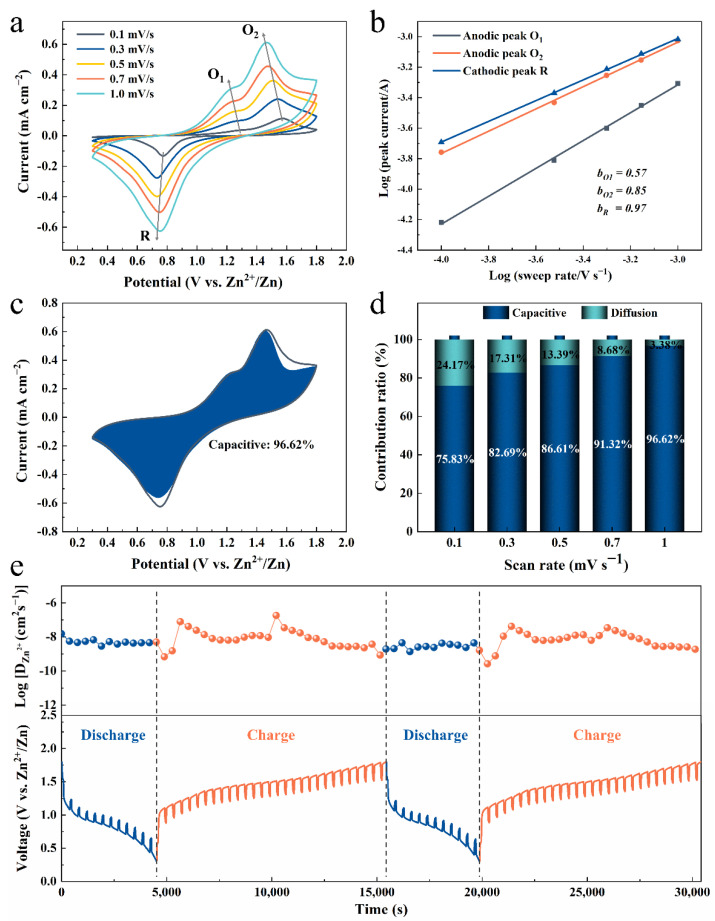
Diffusion kinetics characterization of PANI cathode. (**a**) CV curves of PANI electrode at different scan rates. (**b**) log(*v*) vs. log(*i*) plots at the peak current. (**c**) Contribution ratio of diffusion-controlled vs. capacitive-controlled capacities obtained at 0.1 mV s^−1^. (**d**) Contribution ratio of diffusion-controlled vs. capacitive-controlled capacities at different scan rates. (**e**) GITT analysis results for PANI electrode and corresponding Zn^2+^ diffusion coefficient.

To comprehensively understand the energy storage kinetics of the Zn/PANI batteries, CV curves at various scan rates are shown in Figure 4a. With the increased scan rates from 0.1 mV s^−1^ to 1 mV s^−1^, the CV curves keep similar shapes with subtle shifts in redox peaks, indicating a fast and stable Zn^2+^ adsorption/desorption process even at the high scan rates. Their peak currents (*i*) and scan rates (*v*) have a relationship [6,13,24,25]: *i* = *av^b^*, where *a* and *b* are adjustable parameters. When the value of *b* is close to 0.5, the reaction process relies on the control of ionic diffusion processes. When the value of *b* reaches 1, the corresponding electrochemical behavior is controlled by capacitance. According to the slopes of the log(*i*) vs. log(*v*) plots of all peaks in Figure 4b, the calculated *b* values for peaks O_1_, O_2_, and *R* are 0.57, 0.85, and 0.97, respectively. The value of peak O_1_ is very close to 0.5, which is mostly dominated by diffusion-controlled capacitance. While peak O_2_ and R imply that the surface-dominated pseudocapacitance contribution plays a major role in the following charge storage stage. Along with the increase in scan rate, the capacitive contribution increases and finally reaches to about 96.62% at a scan rate of 1 mV s^−1^ (Figure 4c,d). The large capacitive contribution at low sweep rates suggests a unique pseudocapacitive effect, which can be attributed to the unique surface-dominated reaction. This kind of capacitive-dominated behavior further indicates that fast electrochemical kinetics can match fast surface reactions. Galvanostatic intermittent titration technique (GITT) measurements were further carried out to reveal the kinetics of Zn^2+^ diffusion in PANI electrodes during the cycles. The discharge/charge curves and corresponding diffusion coefficient of Zn^2+^ (D) in GITT measurement for PANI electrodes during the cycles are shown in Figure 4e. The details of the diffusion coefficient calculation are shown in Supplementary Materials. The calculated D values of PANI cathode ranges from 6.25 × 10^−9^ to 7.82 × 10^−8^ cm^−2^ s^−1^ during the two discharge processes and 7.69 × 10^−10^ to 1.81 × 10^−7^ cm^−2^ s^−1^ during the charge processes, which is one or two orders of magnitude higher than other reported manganese oxide and vanadium oxide cathode materials for ZIBs (Table 1) [9,11,13,15,17,25,26,27,28,29,30,31,32]. This result demonstrates that the diffusion kinetics of Zn^2+^ through PANI is quicker and easier, which may be attributed to lower number of electrostatic interactions between Zn^2+^ and host sites reduced by the 3D conductive network’s morphology. PANI also boosts the electrical conductivity of the electrode, allowing for sufficient electrical charge transfers to accommodate the rapid diffusion of Zn^2+^ in the electrode.

**Figure 5:**
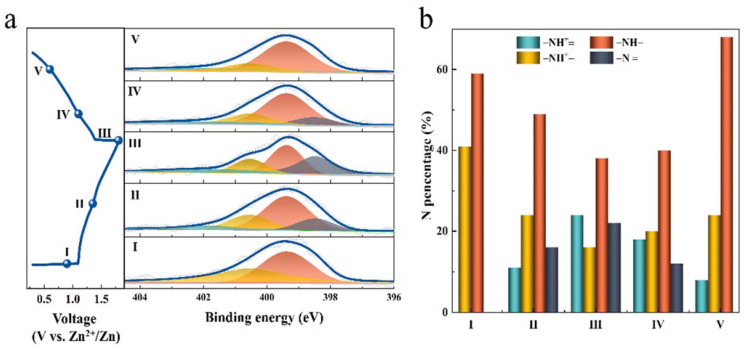
Structure evolution of PANI electrode during cycling. (**a**) Evolution of ex situ N 1s XPS spectra during the charge/discharge process labeled as I−V in the left panel. (**b**) The calculated N contents from N 1s XPS.

To further understand the charge storage mechanism of PANI during the reversible redox reaction, ex situ XPS of N 1s analyses (Figure 5a) were performed on a PANI cathode at different charge/discharge voltages. The N 1s XPS spectra of fully charged PANI cathode were fitted with four peaks related to non-protonated amine −NH−, protonated amine −NH^+^−, non-protonated imine −N=, and protonated imine −NH^+^=, located at 399.38 eV, 400.53 eV, 398.48 eV, and 401.79 eV, respectively. The XPS of S 1s (Supplementary Figure S3) can be divided into two pairs of characteristic peaks, SO_4_^2−^ and SO_3_^−^. During the charge process (from I to III), the peak intensities of −NH^+^= at 401.79 eV and −N= at 398.48 eV are strengthened gradually, whereas in the following discharge process (from III to V), the peak intensities are weakened and ultimately recovered to the original state, which is arising from the reversible reactions between protonated and non-protonated PANI. After full discharge, the XPS analysis’ results in point I show only two components of −NH− and −NH^+^− with the proportion of 59% and 41%, respectively (Figure 5b). When the battery charges from the initial 0.3 to 1.8 V (from I to III), the intensity of −NH− and −NH^+^− decreases while the intensity of −NH^+^= and −N= increases, as a result of the protonation process. The N 1s XPS spectrum of fully charged PANI cathode is fitted with four peaks related to −NH− (38%), −NH^+^− (16%), −N= (22%), and −NH^+^= (24%), respectively. Generally, the former one N signal is referred to the reduced state, while the last three N signals correspond to the oxidized state. While SO_4_^2−^ increases to balance the charge and the −SO_3_^−^H^+^ external dopant PANI cathode is charged to 1.8 V (state III), the amount of oxidized state increases and reduced state decreases. In the oxidation process, the oxidation of the non-protonated components is more facile than that of the protonated −NH^+^− due to an easier loss of electrons for the former.

**Figure 6:**
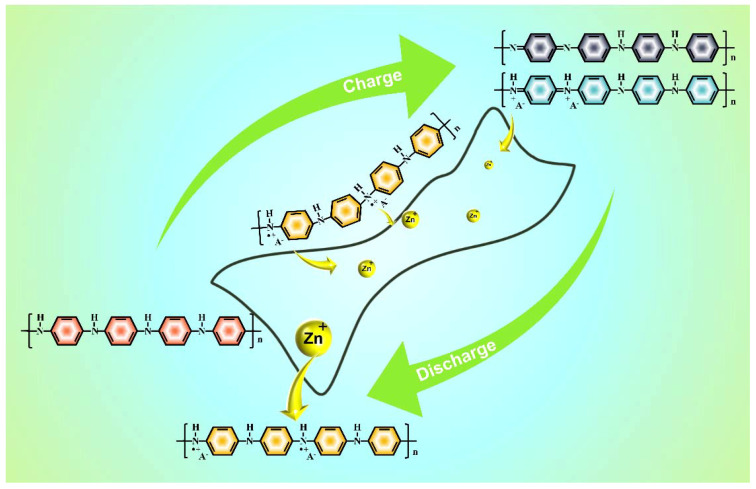
Diagram showing the sequential transformation of protonated and non-protonated PANI. Zn^2+^ adsorption and desorption at the active sites supplied by protonated amine and imine during the process.

Based on the above analysis, we propose a transformation process of PANI electrodes and adsorption/desorption mechanism of Zn^2+^ (Figure 6). In the charging stage, with increases in oxidation voltage, the non-protonated −NH− becomes oxidized to −NH^+^−. Then, −NH^+^− further oxidized with respect to −NH^+^= and −N=. This phenomenon is consistent with the connected double oxidation peaks in the CV curve (Figure 3a). The first oxidation step may be the main contribution to the first small oxidation peak, and the latter oxidation reaction is described by the second oxidation peak. As for discharge processes (from III to V), the peaks of N 1s spectra will go through opposite changes in their intensities compared to the charge process due to the reduction of −NH^+^−, −N=, and −NH^+^=. During the reduction reaction, H^+^ can be consumed to encourage −N= to transform into −NH^+^−, together with the external doping of SO_4_^2−^ to balance the charge. The leftover OH^−^ results in the formation of basic zinc sulfate by a similar manner observed in the previous demonstration [12]. In this process, protonated amine and protonated imine supply abundant active sites for Zn^2+^ ions adsorption and desorption. This sequential transformation can help illuminate the fast diffusion kinetics and energy storage capacities of PANI electrodes.

## 4. Conclusions

We have comprehensively investigated zinc ion storage behaviors in three-dimensional conductive-network-structured PANI. The energy storage mechanism of PANI exhibits four types of N changes during protonated and non-protonated process. The PANI cathode ZIBs shows a high capacity of 74.25 mAh g^−1^ at 300 mA g^−1^ and maintains 48.4% of its initial discharge capacity after 1000 cycles. A corresponding kinetic analysis demonstrated that the diffusion coefficients of PANI are within the range of 6.25 × 10^−9^ to 7.82 × 10^−8^ cm^−2^ s^−1^ for discharge processes and 7.69 × 10^−10^ to 1.81 × 10^−7^ cm^−2^ s^−1^ for charge processes, and the values are one or two orders of magnitude higher than other reported cathode materials. The analysis revealed in this work provides new ideas for understanding the roles of PANI intercalated in host materials. It shows high reference values for active materials using conductive polymers as intercalators to develop energy storage devices with high energy density and stable performance.

## Figures and Tables

**Table 1:** Diffusion coefficient of Zn^2+^ in referenced cathode materials.

Active Materials	Electrolyte	Diffusion Coefficient (cm^−^^2^ s^−^^1^)	Reference
V_2_O_5_@CNTs	1 M ZnSO_4_	10^−10^~10^−8^ (Discharging) 10^−12^~10^−8^ (Charging)	[9]
V_2_O_5_	2 M ZnSO_4_	1.32 × 10^−12^ (Discharging) 3.82 × 10^−11^ (Charging)	[11]
V_2_O_5_·nH_2_O	2 M ZnSO_4_	2.4 × 10^−9^ (Discharging)	[13]
PANI−VOH	3 M Zn(CF_3_SO_3_)_2_	10^−16^~10^−13^ (Discharging) 10^−14^~10^−13^ (Charging)	[15]
V_2_O_5_	ZnSO_4_	10^−11^~10^−9^ (Discharging)	[17]
MnVO/VOH	3 M Zn(CF_3_SO_3_)_2_	3.22 × 10^−12^~(Discharging) 1.46 × 10^−12^~(Charging)	[25]
Mn_0.15_V_2_O_5_·nH_2_O	1 M Zn(ClO_4_)_2_	10^−12^~10^−10^ (Discharging)	[26]
Graphene Scroll Coated α-MnO_2_	2 M ZnSO_4_ 0.2 M MnSO_4_	10^−17^~10^−12^ (Discharging)	[27]
MnO_2_ nanospheres	2 M ZnSO_4_ 0.2 M MnSO_4_	10^−15^~10^−12^ (Discharging)	[28]
δ-MnO_2_	3 M ZnSO_4_ 0.15 M MnSO_4_	10^−13^~10^−9^ (Discharging) 10^−11^~10^−9^ (Charging)	[29]
(NH_4_)_2_V_10_O_25_·8H_2_O	3 M Zn(CF_3_SO_3_)_2_	10^−10^~10^−9^ (Discharging)	[30]
V_5_O_12_·6H_2_O (VOH)	3 M Zn(CF_3_SO_3_)_2_	10^−11^~10^−10^ (Discharging)	[31]
K_2_V_8_O_21_	2 M ZnSO_4_	1.99 × 10^−11^~2.23 × 10^−10^ (Discharging)	[32]
PANI	2 M Zn(CF_3_SO_3_)_2_	6.25×10^−9^~7.82 × 10^−8^ (Discharging) 7.69×10^−10^~1.81 × 10^−7^ (Charging)	This work

## Data Availability

Not applicable.

## Supplementary Materials

The following supporting information can be downloaded at: https://www.mdpi.com/article/10.3390/nano12091438/s1, Figure S1: X-ray diffraction pattern of PANI film electrode; Figure S2: XPS survey scans of the PANI films in the binding energy range of 0–1300 eV. Figure S3: S 1s XPS spectra of the PANI films during the charge/discharge process Supporting Notes: Calculated details of the GITT.
