# Supplementary material for: Zinc-Ion Storage Mechanism of Polyaniline for Rechargeable Aqueous Zinc-Ion Batteries
Source: Nanomaterials (Basel). 2022 Apr 23;12(9):1438. doi: 10.3390/nano12091438 (PMC9103876; doi:10.3390/nano12091438)
Supplement: Supplementary file 1 [file nanomaterials-12-01438-s001.zip › nanomaterials-1623503-supplementary.pdf]

nanomaterials

Supporting Information

Zinc-Ion Storage Mechanism of Polyaniline for Rechargeable Aqueous Zinc-Ion Batteries

Jiangfeng Gong [1,*], Hao Li [1], Kaixiao Zhang [1], Zhupeng Zhang [1], Jie Cao [1], Zhibin Shao [1], Chunmei Tang [1,*], Shaojie Fu [2], Qianjin Wang [2] and Xiang Wu [3,*]

[1] College of Science, Department of Physics, Hohai University, Nanjing 210098, China; lihao5799@163.com (H.L.); kxzhang@126.com (K.Z.), zzp18752006001@163.com (Z.Z.); caojie@hhu.edu.cn (J.C.); zbshao@hhu.edu.cn (Z.S.)
[2] National Laboratory of Microstructures, Nanjing University, Nanjing 210093, China; fushaojie@nju.edu.cn (S.F.); qjwang@nju.edu.cn (Q.W.)
[3] School of Materials Science and Engineering, Shenyang University of Technology, Shenyang 110870, China
* Correspondence: jfgong@hhu.edu.cn (J.G.); cmtang@hhu.edu.cn (C.T.); wuxiang05@sut.edu.cn (X.W.)

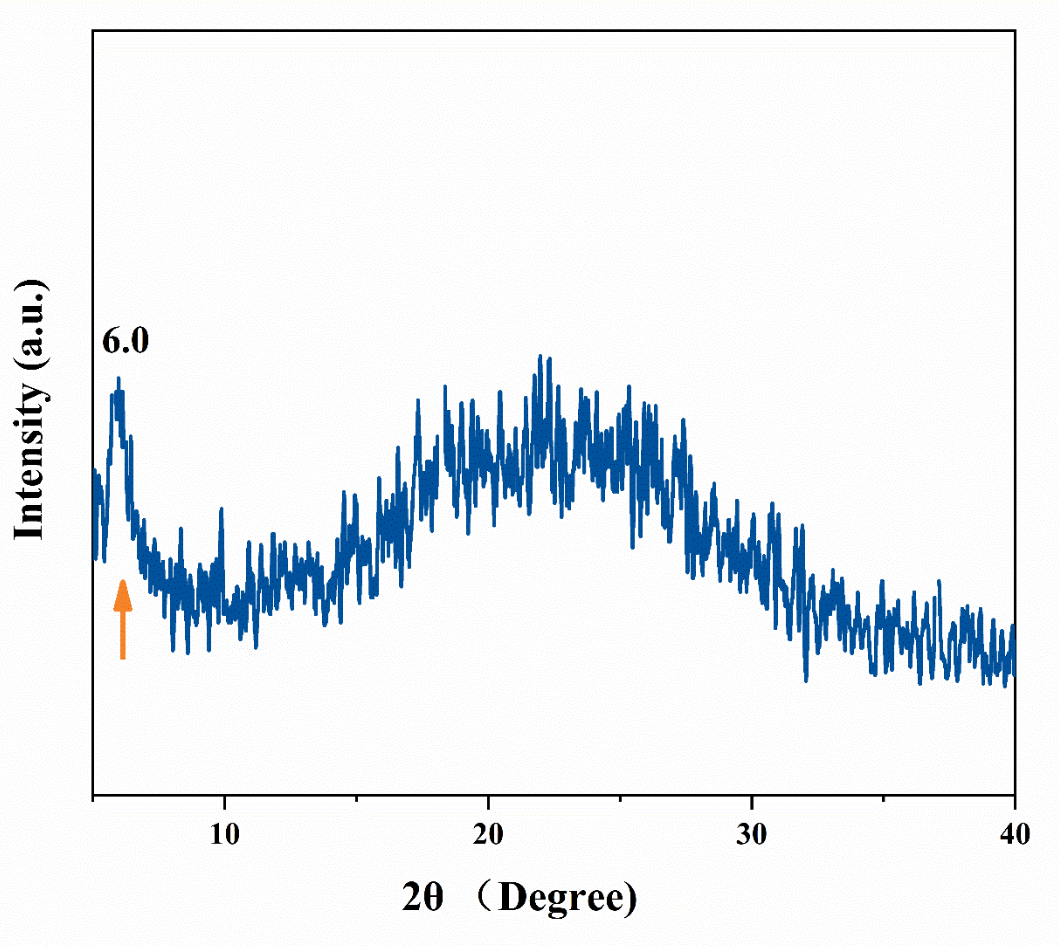

Figure S1. X-ray diffraction pattern of PANI film electrode.

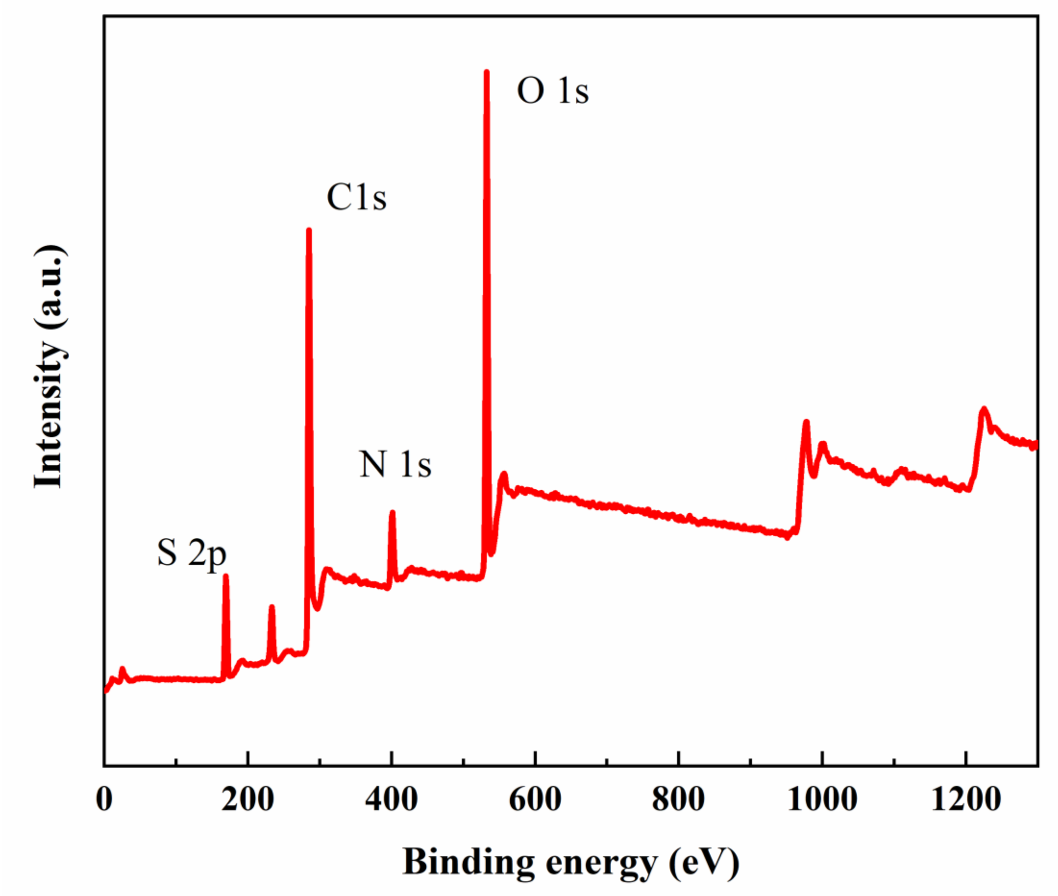

Figure S2. XPS survey scans of the PANI films in the binding energy range of 0–1300 eV. The main peaksfor the elements detected in the films are identified.

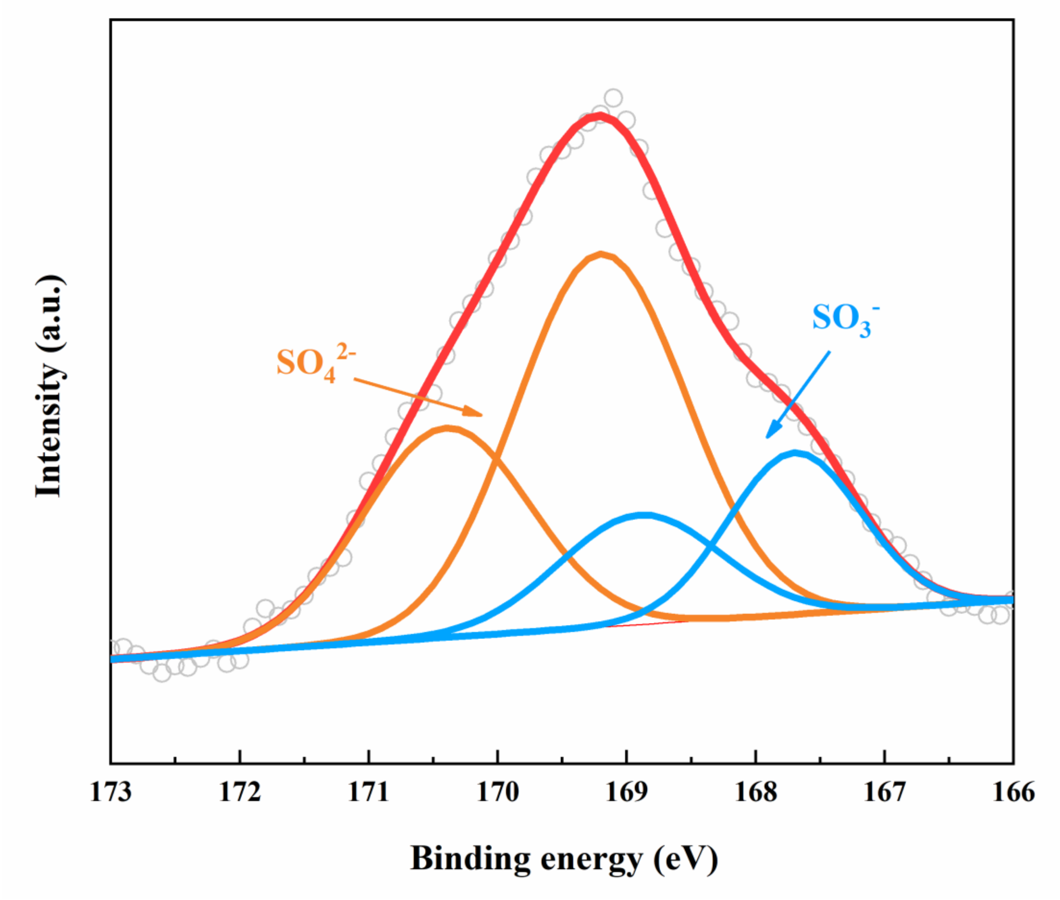

Figure S3. S 1s XPS spectra of the PANI films during the charge/discharge process.

Supporting Notes

Calculation details of the Galvanostatic Intermittent Titration Technique (GITT):

GITT analysis was applied to determine the Zn^{2+} ion diffusion coefficients ($D_{Zn^{2+}}$, $cm^2\ s^{-1}$), following the methodology described in refs. In GITT analysis, the transient voltage that is generated due to the application of a current pulse is monitored as a function of time. Before the GITT measurement, the assembled cell was first discharged/charged at 0.3 A g^{-1} for 30 cycles to obtain a stable state. Subsequently, a galvanostatic pulse (charge or discharge) of 3000 sec at a current density of 50 mA g^{-1} was followed by 60 sec at open circuit step to allow relaxation back to equilibrium. In general, pulse times range from 10 minutes to several hours, depending on the material and its kinetics. Electrode materials with fast reaction rates, i.e. with high diffusion coefficients require shorter time pulse times so as to avoid parasitic side reactions during the hold, once the primary redox reaction is complete. Because of the fast charge behavior of the PANI, 3000 sec pulse duration and relaxation time ware chosen. This was repeatedly applied until the discharge (charge) voltage reached 0.3 V (1.8V) vs. Zn.

$$D_S = \frac{4}{\tau\pi}\left(\frac{n_M V_M}{S}\right)^2 \left(\frac{dE_s}{dE_\tau}\right)^2$$

Where τ is the constant current pulse duration (3000 sec); n_M and V_M are the moles (mol) of PANI and molar volume ($cm^3\ mol^{-1}$), respectively. S is the electrode electrolyte interface area (cm^2) taken as the geometric area of the electrode; dE_s and dE_τ are the change in the steady state voltage and overall cell voltage after the application of a current pulse in a single step GITT experiment.
